# Supplementary material for: An open-sourced, web-based application to analyze weekly excess mortality based on the Short-term Mortality Fluctuations data series
Source: PLoS One. 2021 Feb 5;16(2):e0246663. doi: 10.1371/journal.pone.0246663 (PMC7864412; doi:10.1371/journal.pone.0246663)
Supplement: S1 Table — The table describes the time horizon the data is available for each country. (PDF) [file pone.0246663.s001.pdf]

S1 **Table. The Short-term Mortality Fluctuations data series coverage of 38 countries and regions.**

Country	Period
Australia DCD*	2015-2020
Austria	2000-2020
Belgium	2000-2020
Bulgaria	2010-2020
Canada	2010-2020
Chile	2016-2020
Croatia	2001-2020
Czech Republic	2005-2020
Denmark	2007-2020
England and Wales	2010-2020
Estonia	2000-2020
Finland	1990-2020
France	2000-2020
Germany	2016-2020
Greece	2016-2020
Hungary	2000-2020
Iceland	2000-2020
Israel	2000-2020
Italy	2015-2020
Latvia	2000-2020
Lithuania	2000-2020
Luxembourg	2000-2020
Netherlands	1995-2020
New Zealand	2010-2020
Northern Ireland	2015-2020
Norway	2000-2020
Poland	2000-2020
Portugal	2000-2020
Republic of Korea	2010-2020
Russia	2000-2019
Scotland	2000-2020
Slovakia	2000-2020
Slovenia	2000-2020
Spain	2000-2020
Sweden	2000-2020
Switzerland	2000-2020
Taiwan	2000-2020
United States of America	2013-2020

Table notes *Australia DCD (doctor certified deaths) includes the provisional counts of deaths occurred in Australia, and this series is not comparable with other total death counts.
