# Supplementary material for: An open-sourced, web-based application to analyze weekly excess mortality based on the Short-term Mortality Fluctuations data series
Source: PLoS One. 2021 Feb 5;16(2):e0246663. doi: 10.1371/journal.pone.0246663 (PMC7864412; doi:10.1371/journal.pone.0246663)
Supplement: Attachment — Submitted filename: responsetoreviewers.pdf [file pone.0246663.s002.pdf]

Response to reviewers

Dear Editor, Dear Reviewers,

below, please find our detailed response to the comments and suggestions for which we are grateful and we think these were extremely helpful for us to improve the quality of our manuscript. We acknowledge that the crucial part of the manuscript is the one detailing the calculations of excess mortality and, therefore, we extensively revised the 'Materials and methods' section. Based on your comments we extended both the 'Introduction' and 'Discussion' that were rather short in the previous version of the manuscript. Where possible, we collected and organized the comments into sections with our corresponding response after a short divider.

Editor: This is a very important paper and contribution to the field and public health planning. The paper reads well and informs the reader about the database and how one is supposed to use it.

Reviewer 1: This submission provides an overview of the new Short-term Mortality Fluctuations data series, available at the Human Mortality Database, and a new tool for visualizing excess mortality. The paper is very informative and easy to understand, even for non-technical users.

Reviewer 2: This is really great and I'm very thankful the teams at Max Planck and Berkeley have put this incredible resource together. As a long time user of the HMD, I just want to first say thank you! ... Again, thank you for providing such valuable resources!

Reviewer 3: This article presents a novel tool for visualizing weekly excess mortality in 36 countries. Excess mortality provides us with valuable information during an epidemic or other natural or man-made disasters. In the context of the global COVID-19 pandemic, excess mortality estimations have been invaluable to evaluate the impacts of the pandemic and to compare territories. The tool presented is simple, yet it could be of vital importance to examine and compare the evolution of mortality in this and in other health emergencies and disasters worldwide. I really appreciated the simplicity and speed of the tool (other shiny apps can be quite slow). I think the article is interesting for a wide range of readers (researchers, demographers, public health professionals, policymakers etc.) and is suitable for publication in this journal.

We would like to thank all of you for the appreciation of our manuscript and the very encouraging words.

Editor: There are also some minor adjustments in language and several typos across the paper.

Reviewer 1:

- Main title, lines 113 and 126: You're juggling around with the terms "excess mortality" vs. "mortality excess". As the term "excess mortality" is commonly used in epidemiological studies, you should stick with it or, if applicable, "excess deaths".
- Line 7: please delete the URL from the brackets and stick to the common citation style of internet sources.

- Line 8: The "STMF" abbreviation should be put before "data series", not after, as that is not included in the abbreviation.
- Lines 10-11: Please delete the country listing. Instead, I would like to see a table in the appendix, which contains a line for each country with the information, for which time horizon the data is available in each country. This varies significantly between the countries and you could give for the users a quick overview, which would be useful for comparative international studies. This table would be a nice addition to the description you provide here.
- Line 13: Add an "s" to "resource".
- Line 43: Change "These appear" to "This appears"

Reviewer 2: 2) There are a few minor typos throughout.

Thank you very much for these comments. We carefully revised the text according to your suggestions. Please note, the revision with the additional text in the manuscript (detailed in our response later) caused some blocks of texts being moved, dropping the 'over the period' from variable names in the application, reordering the equations in the text for better transparency and readability. We added a table with the country listing to the Supporting Information (lines 23, 248-249).

Reviewer 1: I would like to know in the discussion, why you don't also provide weekly data on cause-specific mortality and whether you plan to do so eventually. I know there's a report on the HMD homepage where you mention that as well. Please discuss this topic shortly in this contribution as well.

Methods for assessment of the excess mortality purposely avoid usage of data on causes of death. This is to make sure that the resulting excess mortality estimates are comparable across space and time and do not depend on differences in principles and practices of cause-of-death diagnostics and coding. However, this does not mean that the analysis of weekly mortality by causes of death is of no interest. Although, it should be carried out with special care, including the participation of country specialists with sufficient knowledge of cause-of-death statistics in different countries and time periods. We expanded the Introduction (lines 2-18) and the Discussion (236-247) with more information on cause-specific mortality.

Editor: The main one is related to the calculation of excess mortality - please provide more detail.

Reviewer 2: 1) I'd like to see the authors include the calculations they are using for excess mortality in this manuscript.

Reviewer 3: Major 2. As excess mortality is the main measure presented in the paper, further description of the different estimation methods (and why you choose the ones presented in the paper) is needed. For example, why did you decided to choose the average of the reference

period and not the maximum historical value? What is the recommended reference period? Why eliminate winter months but keep summer months of years with clear outliers as European countries in 2003? A brief explanation on the methods will really help the reader to understand these questions.

Major 3. When explaining the reference levels, you define a summer and winter seasons based on calendar weeks. This definition does not fit for countries in the southern hemisphere. Please correct or explain.

In the web application, we provide users with a few simple and intuitively transparent methods for estimation of the intra-annual mortality excess. Interpretation of their outcomes is also straightforward. Our purpose was to provide a toolkit for a simple preliminary analysis. A further in-depth examination of the data should be done by the users themselves according to specific research purposes using the STMF data files.

The Reviewers' comments suggest a need for a more detailed explanation of the excess mortality assessment. In general, there are two major approaches to the estimation of the intra-annual mortality excess [1,2]. The first (and a more traditional) one is focused on the variation of mortality across weeks or months within a year in question and expresses a notion of "seasonality" [3–6]. The second one (actively used for assessment of the COVID-19 related mortality), looks at mortality deviations for certain weeks/months compared to the mortality experience of previous years for the same weeks/months [2, 7–9].

Equations 1, 2 and 3 correspond to the mainstream approach (the just mentioned second approach) that compares the observed week-specific mortality with that during a certain reference period. Correspondingly, the reference level of mortality varies across weeks. Equation 1 determines the reference level as the average over the reference period (following examples of the excess mortality monitorings by the ONS in England and Wales, the New York Times, the Finacial Times, the Our World in Data, and many others). In some countries (especially countries of Eastern Europe) mortality was steeply decreasing over the last 15-18 years. To address these trends, equation 2 determines the reference mortality level as a continuation of the week-specific trends. According to mainstream practice by the ONS and others, the reference period may include several years (5-7 years) preceding the target year. For example, the years 2015-19 with 2020 as a target year. However, depending on purpose, the reference period can be longer or shorter and may also include the target year (e.g. interpolation instead of extrapolation).

Equations 1 and 2 provide two widely used ways for assessment of excess mortality compared to previous years. However, researchers might prefer to use reference levels based on maximal or minimal values over the reference period or on Fourier time series instead of week-specific averages or linear trends. To achieve it, they will have to calculate those from the STMF data themselves.

Equations 4, 5 and 6 correspond to the "seasonality" approach. The excess mortality expresses losses due to mortality difference between different periods within a year. Here, the reference level

of mortality is a week-independent constant. In equation 4, the reference level is defined as the average across all 52 weeks. In equation 5, this level is defined as an average of mortality over a lower mortality season (non-winter season). This method may be used with the reference period consisting of the target year only. In this case, the excess mortality expresses the mortality excess compared to the average mortality for all available weeks (equation 4) or to the average mortality over lower-mortality weeks of the target year (equation 5 and 6). However, if weekly mortality experiences large random fluctuations (in countries with small populations), the reference period could include the target year and a few years before this year (or around this year). For example, the years 2014-18 with 2018 as a target year.

In the revised manuscript, we largely use the explanations and justifications that are given above in response to this comment for a better understanding of the "Materials and Methods" and especially equations 1-6.

It is true that in countries of the southern hemisphere the non-winter period should be re-defined. Thank you for noticing this problem. We have modified the Application regarding the definition of the non-winter season in Australia, New Zealand, Chile and also explain it in the revised text of the "Materials and methods" section. A definition of the non-winter season for this group of countries is added now to the "Materials and Methods".

To address the heat-wave outbreaks and other mortality elevations out of the winter season, we added to the Application an additional reference-level option. It allows now to define the reference level of mortality as the average mortality for the lower quartile of the mortality distribution across weeks within the reference period. This is now equation 3 and 6 in the revised text.

> **Reviewer 3:** Major 1. The introduction is too short, and it fails to provide the necessary justification for the tool presented. A brief description of excess mortality and its usefulness for public health and policy will really help to show non-specialist readers the true importance of the data and visualizations provided. A proper contextualization of the COVID-19 pandemic and how excess mortality is helpful in this context can also be important.

Thank you for pointing this out. We expanded the Introduction, with additional text (lines 2-18) and references [2-4] providing more evidence supporting the choice of the excess mortality method.

> **Reviewer 3:** Major 4. Both of the presented estimations (numerical excess deaths and crude death rates) do not allow comparisons between countries (because of population size, mortality trends and socio-demographical characteristics as ageing). This has to be addressed in the paper. Is there a way to compare territories? Can the excess mortality in percentage be useful?

We provide estimates of the mortality excess in terms of death rates for all ages combined or for several broad age groups. These quantities are independent of the population size. It might be

also possible to carry out an age-standardization. But this is a non-trivial exercise for countries without detailed mortality data by week and age. The adjustment can be done in several ways. Therefore, we decided to delegate it to the STMF users. Use of the relative mortality excess = absolute mortality excess/reference mortality level * 100 is a good idea and this (unitless) measure of excess is now added to our Application.

Reviewer 3: Major 5. The discussion is short and I fond that much can be said about the usefulness, limitations and strengths of the tool. Also, the reference to the fertility tool is not well connected to the rest of the article (how the fertility tool relates to short term mortality fluctuations?).

Thank you for drawing our attention to this and we fully agree. We expanded and revised the text for a more detailed discussion and better readability and interconnectedness (lines (215-247).

Reviewer 3: Minor 1. Why did you decided to include the target year in the linear models to estimate reference levels? Please explain.

The excess mortality estimates determined by equations 1, 2 and 3 are flexible and may be based on a reference period that includes the target year. But in most cases, the users might want to define the reference period as 5-7 years preceding the target year. A reference period corresponding to equations 4, 5 and 6 may or may not include the target year or may even include only the target year.

Please, note our response above to your comment Major 2 for more details. These are also included now in the text of the "Materials and Methods".

Reviewer 3: Minor 2. When presenting death rates, it could be better to include the value in death per 10.000 or 100.000 persons/week. The interpretation of decimals can be hard for many readers.

We agree. The revised Application shows now death rates per 100 000 person-years, a more familiar format for many demographers and epidemiologists.

Reviewer 3: Minor 3. The figures presented are low quality and do not reflect the beauty of the web tool.

The low-quality figures are shown only in the pdf file built by the online editorial system of Plos One. In the published article, the figures are included in their original resolution (maybe resized due to the layout). These currently can be downloaded individually via the link provided in the compiled submission pdf in the upper-right corner on the page of each figure.

Reviewer 3: Minor 4. The tool allows to change the colors of the graphs, which is helpful; but when displaying two graphs, it does not allow to change colors of each individual graph. For comparisons, this could be a neat feature.

We added the possibility for the users to change the colors independently. Thank you very much for the excellent idea.

References

[1] Rau R. Seasonality in human mortality: a demographic approach. Springer Science & Business Media; 2006.

[2] Nicoll A, Ciancio B, Chavarrias VL, Mølbak K, Pebody R, Pedzinski B, et al. Influenza-related deaths-available methods for estimating numbers and detecting patterns for seasonal and pandemic influenza in Europe. Eurosurveillance. 2012;17(18):20162.

[3] Sakamoto-Momiyama M. Changes in the seasonality of human mortality: a medico-geographical study. Social Science & Medicine Part D: Medical Geography. 1978;12(1):29–42.

[4] Andreev E, Biryukov V. Influence of influenza epidemics on mortality in Russia (In Russian). Vestnik Statistiki. 1998;(2):73–77.

[5] Healy JD. Excess winter mortality in Europe: a cross country analysis identifying key risk factors. Journal of Epidemiology & Community Health. 2003;57(10):784–789.

[6] Liddell C, Morris C, Thomson H, Guiney C. Excess winter deaths in 30 European countries 1980–2013: a critical review of methods. Journal of Public Health. 2016;38(4):806–814.

[7] Thompson WW, Weintraub E, Dhankhar P, Cheng PY, Brammer L, Meltzer MI, et al. Estimates of US influenza-associated deaths made using four different methods. Influenza and other respiratory viruses. 2009;3(1):37–49.

[8] Nielsen J, Mazick A, Glismann S, Mølbak K. Excess mortality related to seasonal influenza and extreme temperatures in Denmark, 1994-2010. BMC infectious diseases. 2011;11(1):350.

[9] Kontis V, Bennett JE, Rashid T, Parks RM, Pearson-Stuttard J, Guillot M, et al. Magnitude, demographics and dynamics of the effect of the first wave of the COVID-19 pandemic on all-cause mortality in 21 industrialized countries. Nature medicine. 2020; p. 1–10.
